# Supplementary material for: Structural basis for the ligand promiscuity of the neofunctionalized, carotenoid-binding fasciclin domain protein AstaP
Source: Commun Biol. 2023 Apr 28;6:471. doi: 10.1038/s42003-023-04832-z (PMC10147662; doi:10.1038/s42003-023-04832-z)
Supplement: Supplementary file 2 — Supplementary Information [file 42003_2023_4832_MOESM2_ESM.pdf]

-Supplementary data -

Manuscript title:

“Structural basis for the ligand promiscuity of the neofunctionalized, carotenoid-binding fasciclin domain protein AstaP”

Supplementary Tables

Supplementary Table 1. Primers used in this study.

Primer	Sequence
T7_forw	TAATACGACTCACTATAGGG
T7_rev	CGGGCTTTGTTAGCAGCCG
AstaPo1_dN_forw	ATTACATATGTATGCTACGTTGAGCAATGCCG
AstaPo1_dC_rev	AATACTCGAGTCAGGATACCACTCCTGGGG
AstaPo1_W79F_rev	CCAGGATAGTAAATGTAGTGTTTG
AstaPo1_I176F_forw	AGGCAGTGTATTTCATGTCATCAAC
AstaPo1_Q56T_L57F_forw	AGCTCCTACGTTCACGACGTTATTTGCCGCTGTACGTGCGGC
AstaPo1_Q56T_L57F_rev	ACGTCGTGAACGTAGGAGCTGCCGCTCCTGCAGTTACGGC
AstaPo1_Q56T_forw*	CAGCTCCTACGTTGACGACGTTATTTGCCGCTGTACGTGCGGC
AstaPo1_Q56T_rev*	CGTCGTCAACGTAGGAGCTGCCGCTCCTGCAGTTACGGCATTG
AstaPo1_L57F_forw	CTCCTCAGTTCACGACGTTATTTGCCGCTGTACGTGCG
AstaPo1_L57F_rev	AACGTCGTGAACTGAGGAGCTGCCGCTCCTGCAGTTACG

***** According to the DNA sequencing results, the Q56T mutant contained an inadvertent substitution A52V, which mimicked the sequence difference between AstaPo1 and SynAstaP and was included in the analysis.

Supplementary Table 2. Properties of the proteins used in this study.

Protein	$\varepsilon_{280\ nm}$, M^{-1} cm^{-1}	ZEA A_{vis}/A_{UV} *	M_w (kDa)	pI
AstaPo1.WT	22460	2.8	21.6	10.1
AstaPo1.W79F	16960	~3	21.6	10.1
AstaPo1.I176F	22460	2.8	21.6	10.1
AstaPo1.Q56T/L57F	22460	2.8	21.6	10.1
AstaPo1$^{41-223}$	22460	2.8	19.7	10.0
AstaPo1$^{21-190}$	11460	~4	17.8	10.0
AstaPo1$^{41-190}$	11460	~4	15.9	9.9
SynAstaP (P74615)	1490	-	16.3	4.7

All constructs contained GPHM extra residues at their N terminus, which was taken into account in calculations.

*The expected Vis/UV absorbance ratios in ZEA-bound holoforms are indicated assuming the extinction coefficient of AstaPo1-bound ZEA at 463 nm and 280 nm equal to 120,000 M^{-1} cm^{-1} and 20,000 M^{-1} cm^{-1}, respectively, and taking into account protein extinction coefficients at 280 nm.

Supplementary figures

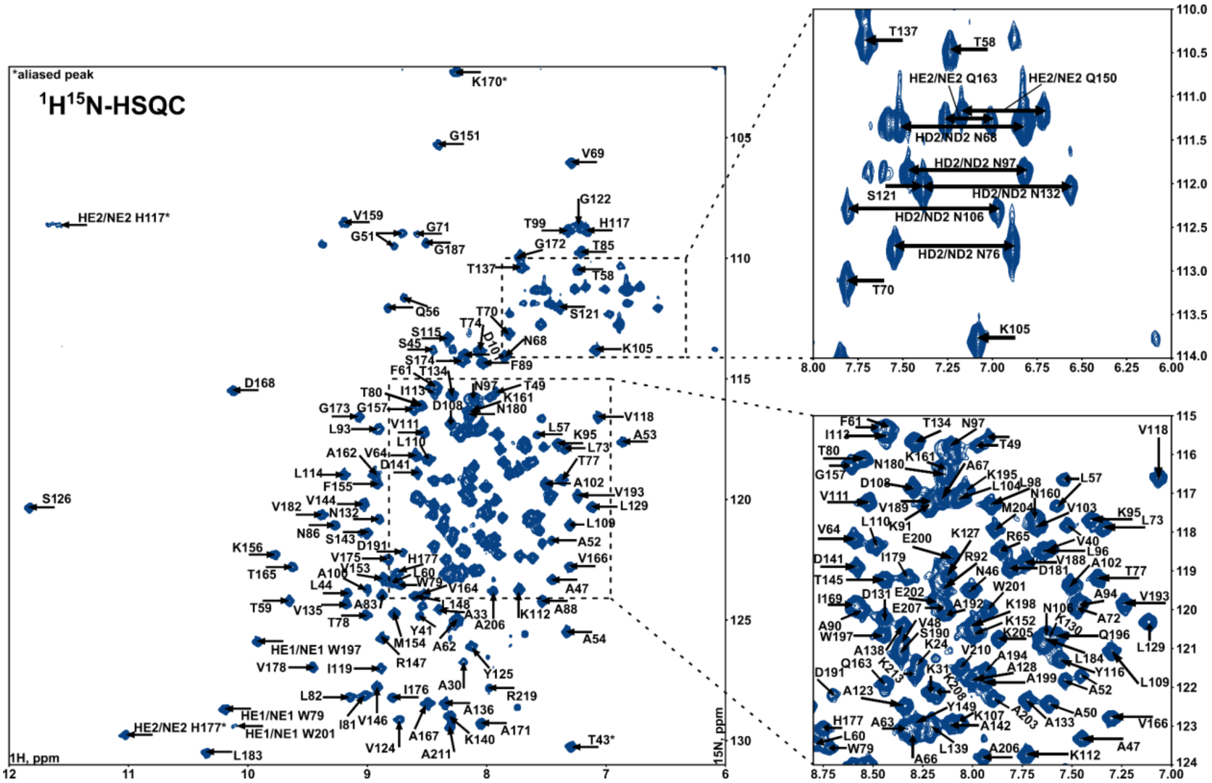

Supplementary Figure 1. $^{1}H^{15}N$-HSQC spectrum of AstaPo1/AXT complex. T=313K, pH=7.5. Positions of aliased peaks of the histidine side chains are highlighted with asterisks.

Supplementary Text 1. Analysis of conformational heterogeneity of AXT in complex with AstaPo1.

To elucidate the source of the conformational heterogeneity observed for the AstaPo1-bound AXT molecule, we considered three possible options: 1) the possibility that AstaPo1 can bind AXT in two alternative orientations, 2) the *s-cis-s-trans* isomerism of the C6-C7 bond and 3) the R-S optical isomers of astaxanthin, which are observed at the position 3 of the β-ionone group (**Supplementary Figure 2**). Inspection of the intermolecular distances suggests that the orientation of the isoprenoid fragment of AXT is the same in the two observed states, while the conformations or configurations of both β-ionone rings are different. To distinguish between the two remaining options, we analyzed the intra- and intermolecular distances and chemical shifts in AXT. Such an analysis reveals that one of the states of AXT corresponds to the (3S,3S)-AXT with the s-*cis* configuration of C6-C7 bonds for both rings. The s-cis conformation of AXT is more favorable [24] and corresponds to the state of the molecule in the DMSO solution (**Supplementary Figure 4**). The conformation of AXT in the second state is much less obvious. First of all, at one side of AXT, which contacts the helix α1, the conformation of the C6-C7 bond is s-cis in both states (the double bond proton, closest to the C17/C16 methyl groups is proximal to the isoprene methyl group). At the same time, the pattern of intermolecular NOEs observed for the H3-proton and C17/C16 methyl groups does not agree with the s-cis configuration of the bond and 3S-configuration of this β-ionone ring (**Supplementary Figure 5**). Thus, the only obvious option that agrees with all the data is the presence of a 3R-isomer of AXT (**Supplementary Figure 5**). According to the literature, both

synthetic and natural AXT exists as a mixture of 3S and 3R isomers in various proportions [25], however, the exact isomer contents for the AXT preparation that we used in our research are not known. Apparently, in our particular case, the isomers are present in equal populations for both the headgroups of AXT. According to the ^{13}Cα chemical shifts (**Supplementary Figure 6**), that are indicative of the protein secondary structure, AstaP adapts to the headgroup configuration by the subtle motions (the chemical shift difference is as small as 0.3 ppm) in the hinge region connecting helices α1 and α2.

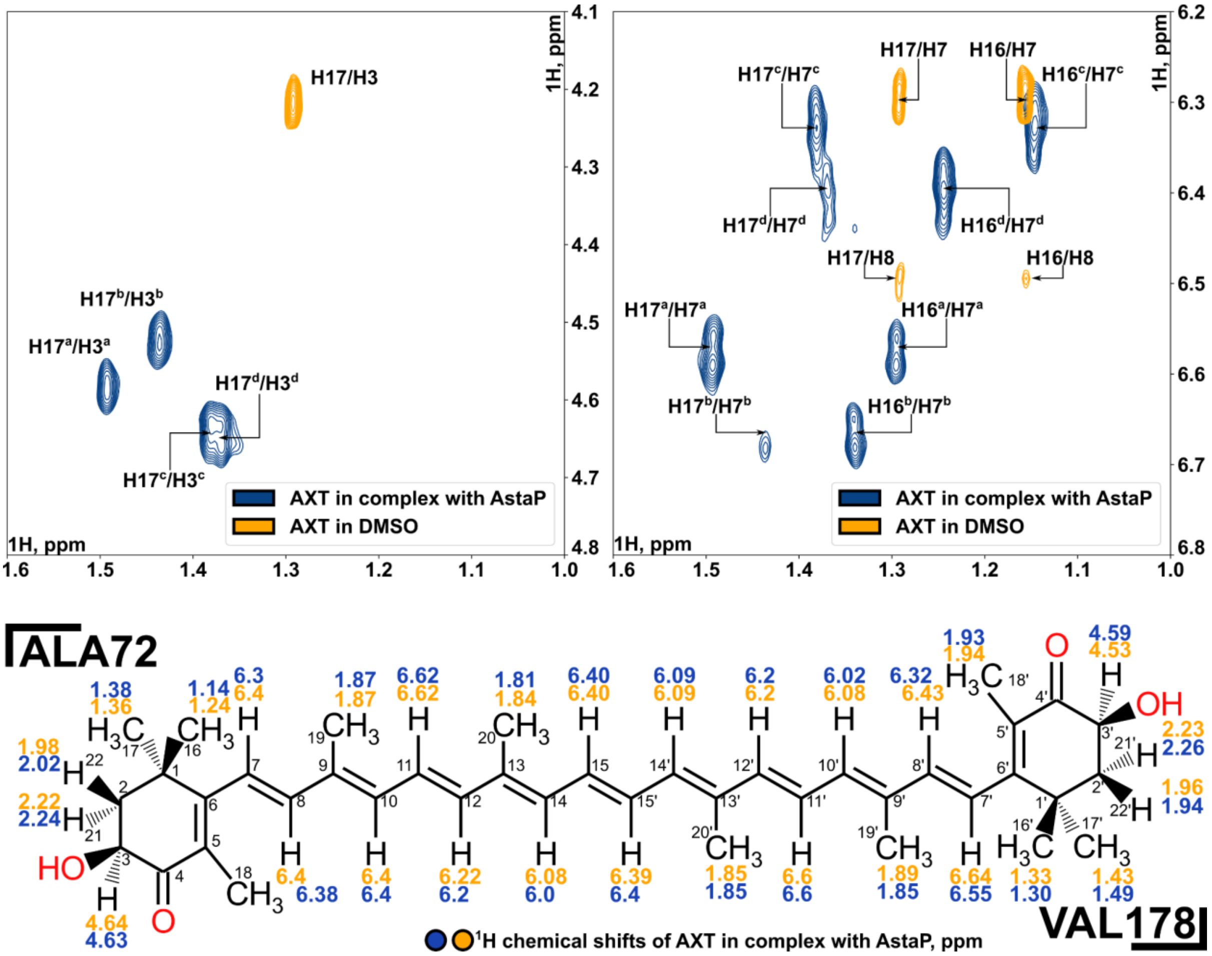

Supplementary Figure 2. Fragments of the 2D ^{13}C/^{15}N-double filtered-NOESY spectra recorded for the AstaPo1/AXT complex in water (shown in blue) and of the 2D-NOESY spectra recorded for the AXT in DMSO (shown in yellow). Four sets of signals corresponding to AXT in complex with AstaPo1 are indicated by the superscripts a-d. The structure of AXT with the atom numbering is also shown. Assignment of the proton chemical shift for the two states of AXT is shown by yellow and blue numbers, Ala72 and Val178 denote the residues that are in contact with the corresponding AXT headgroup in both states, according to the ^{13}C-filtered NOESY experiment.

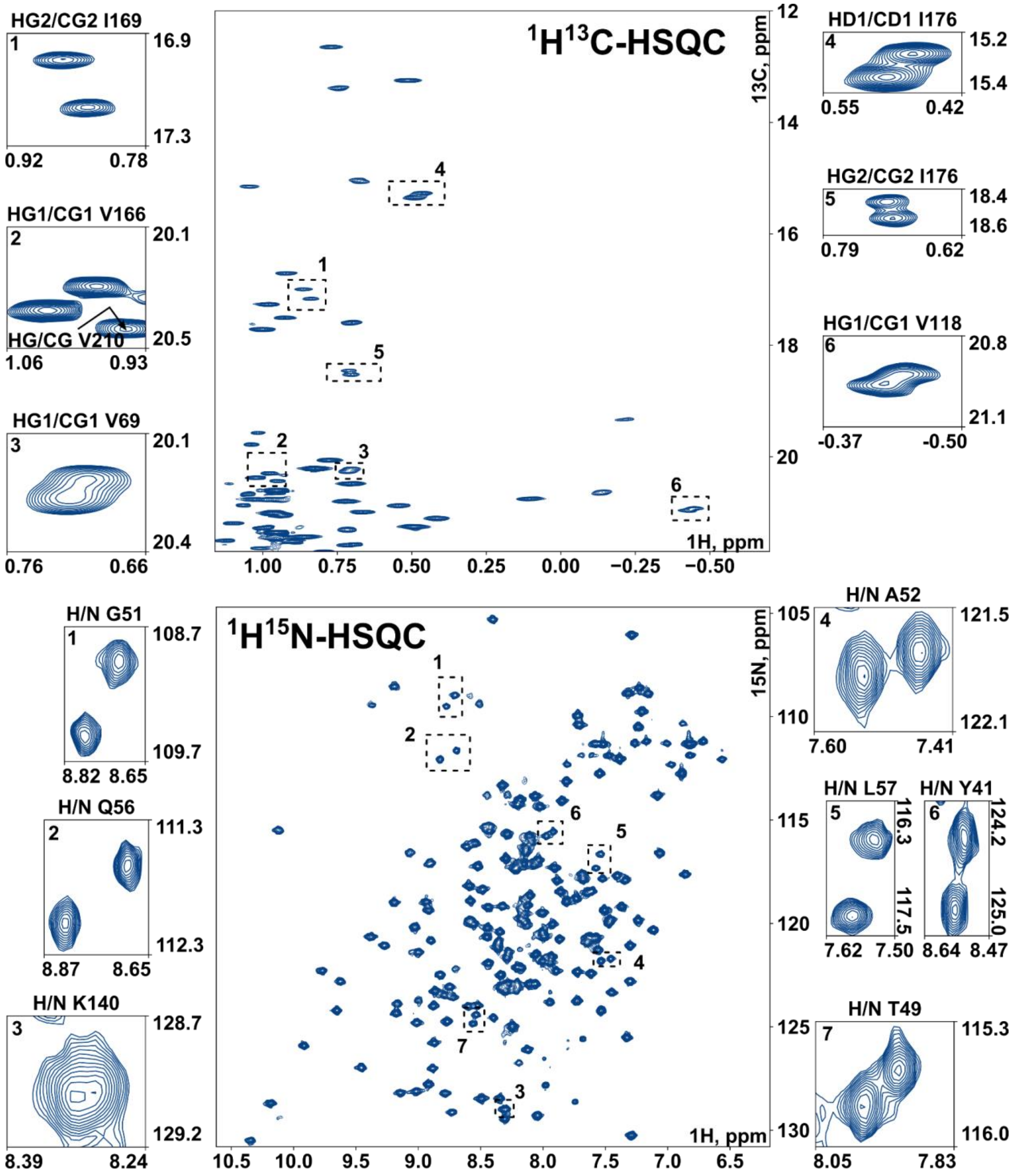

Supplementary Figure 3. Signal splitting in ^{1}H,^{13}C-CT-HSQC (a fragment containing the signals from the Leu, Ile, Val methyl groups) and ^{1}H,^{15}N-HSQC spectra of AstaPo1/AXT complex recorded at 40° C. Split signals are shown by numbered dashed rectangles and are additionally shown at high resolution.

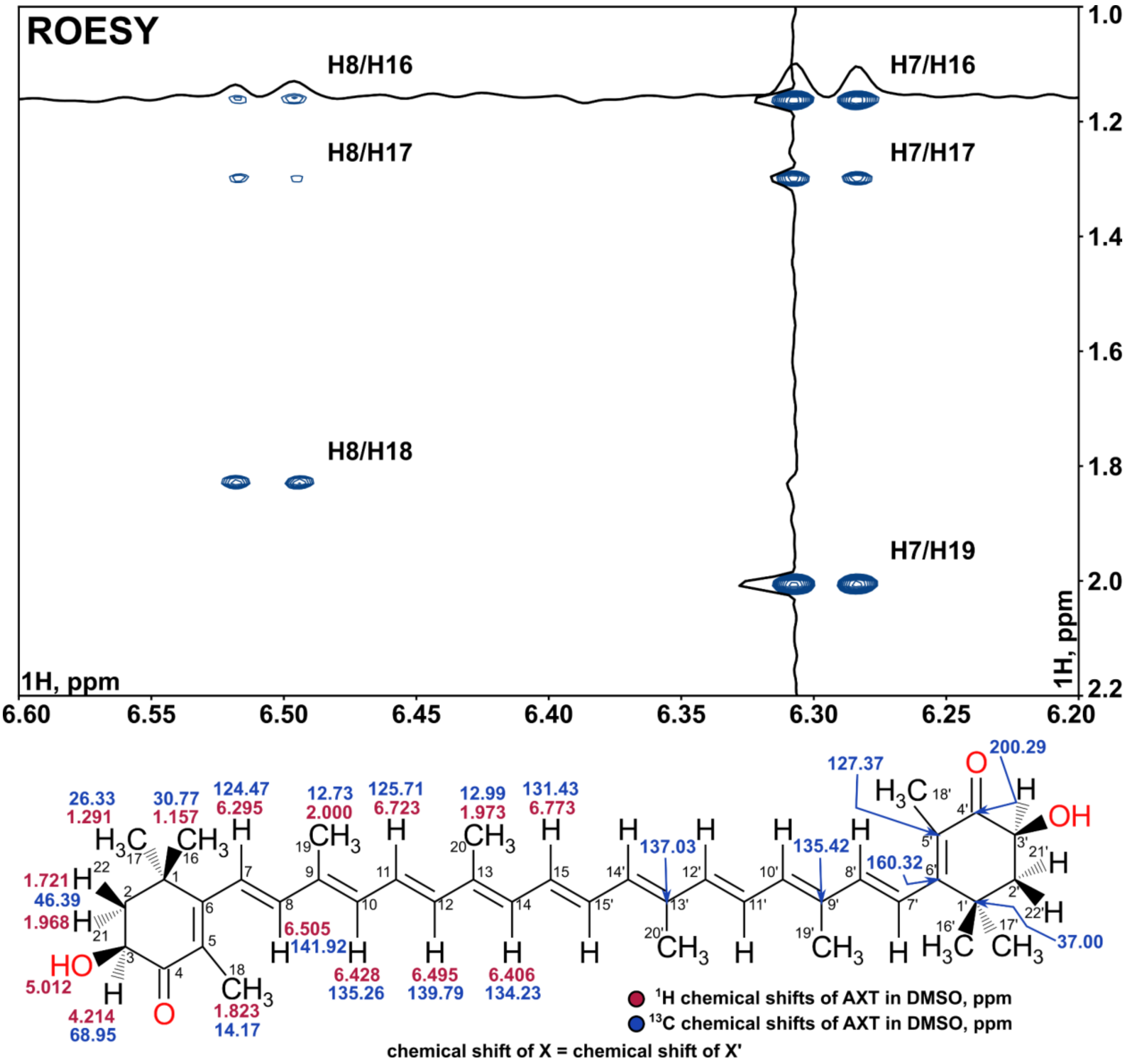

Supplementary Figure 4. A fragment of ROESY spectrum of 1 mM AXT solution in DMSO, showing the contacts between the C1 (H16 and H17), C5 (H18), and C9 (H19) methyl groups in the AXT headgroup and H7/H8 protons of the double bond. The analysis supports the s-cis configuration of the C6-C7 bond. The structure of AXT and chemical shift assignments are shown below.

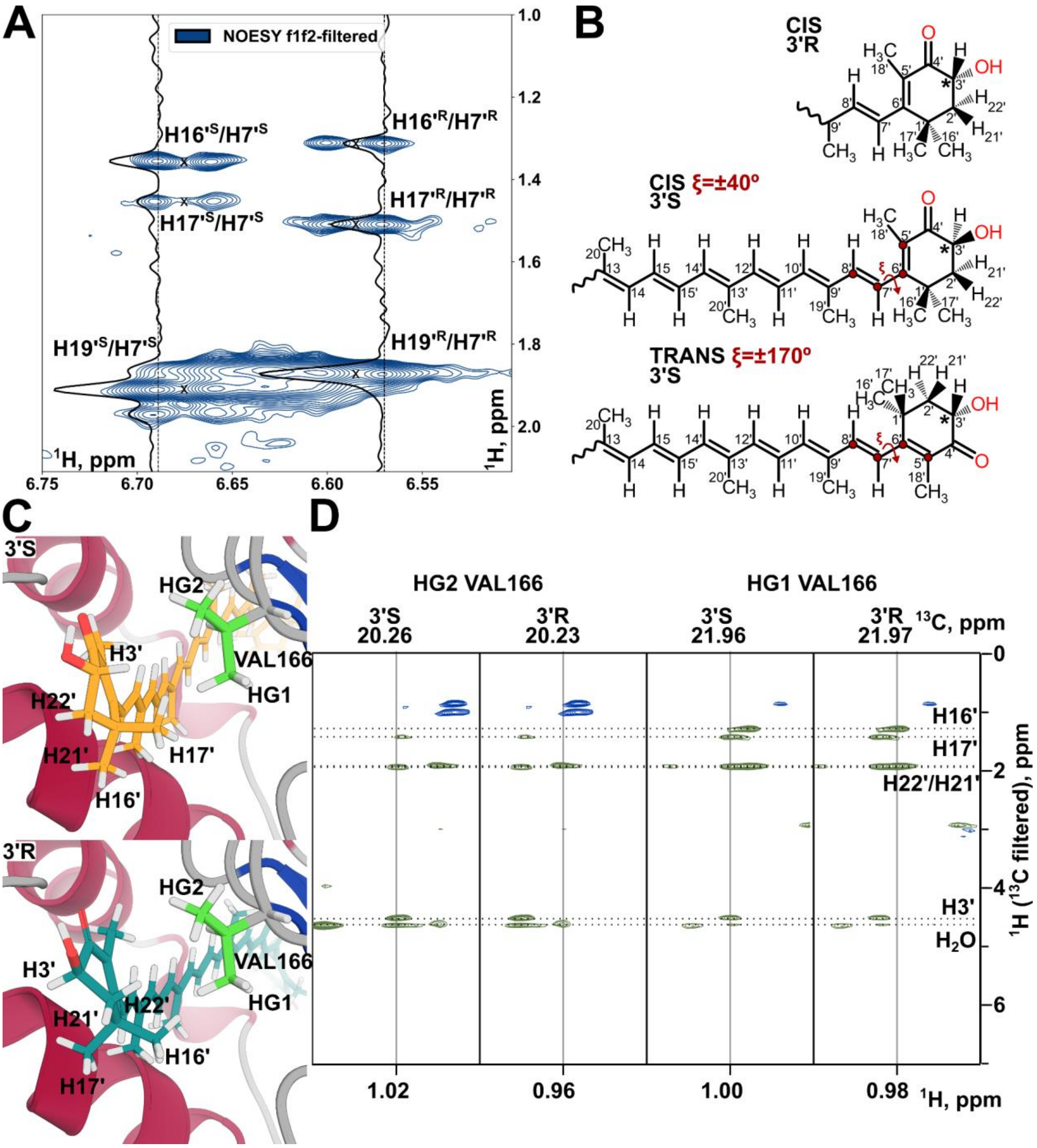

Supplementary Figure 5. Analysis of AXT configurations in complex with AstaPo1. **A** - a fragment of 2D f1f2-filtered NOESY spectrum of AstaPo1/AXT complex, showing the NOE-contacts between H7' and methyl groups (H16', H17' and H19') of AXT. The strongest peak is observed with the H19 methyl group, supporting the *cis* conformation of C6-C7 bond. **B** - Cis/Trans conformations and 3'S/3'R isomers of AXT. The stereocenter is marked with an asterisk, red circles indicate atoms that define a dihedral angle ξ. **C** - 3'S (from the NMR structure) and 3'R (putative model) stereoisomers of AXT in the exit from the binding pocket of AstaPo1 contact the Val166 sidechain. **D** - 2D 1H/1H strips from the 3D 13C/15N-filtered,13C-edited-NOESY-HSQC, showing intermolecular NOE-contacts between HG1/HG2 of VAL166 and atoms of the β-ionone ring of AXT. Contacts to the H3' proton are observed only in the 3'S state.

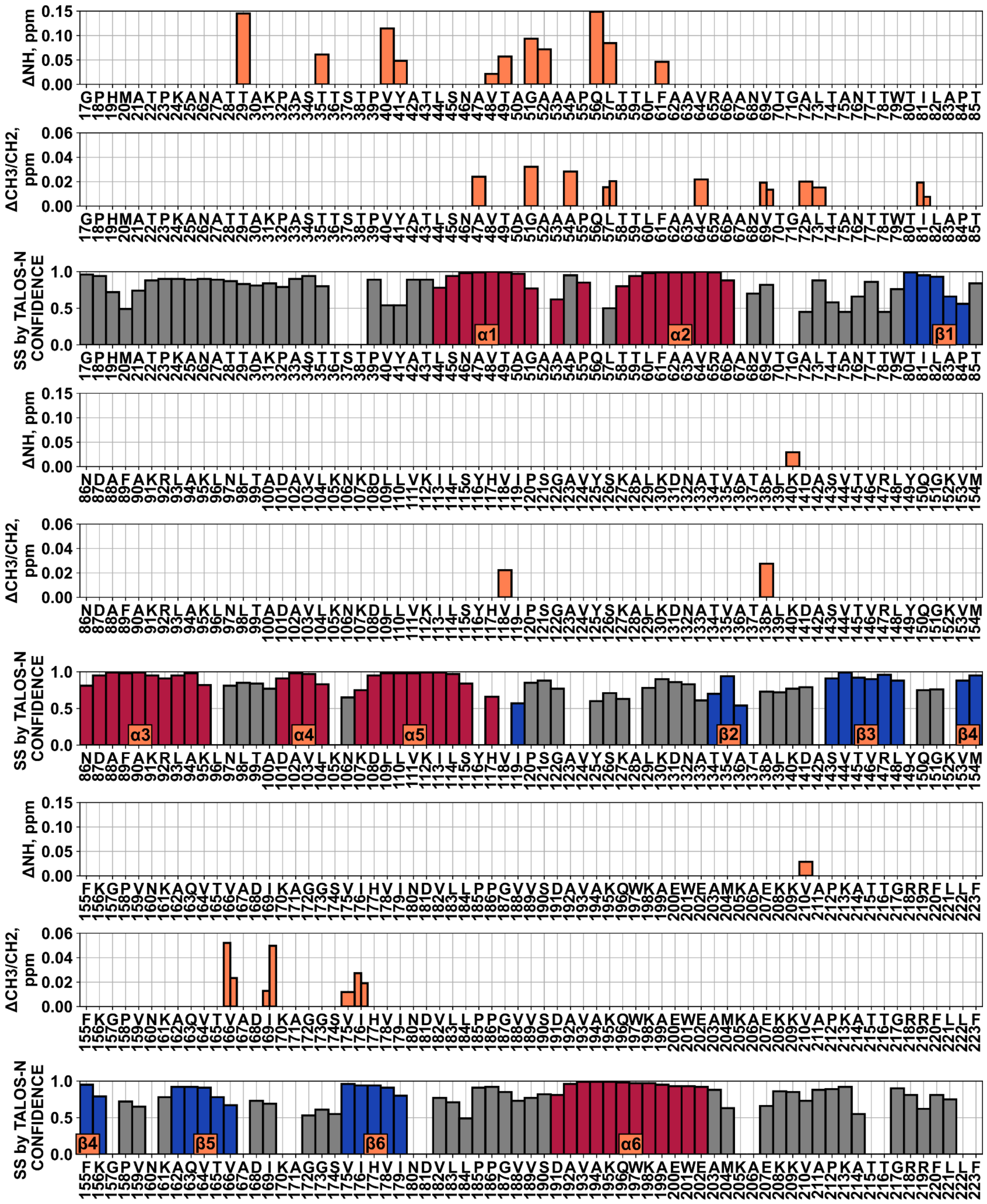

Supplementary Figure 6. Peak splittings in the NMR spectra of AstaPo1/AXT complex. Chemical shift perturbations of AstaPo1 amide and methyl groups that take place due to the conformational heterogeneity of AstaPo1/AXT complex. Additionally, the secondary structure is provided, as predicted by TALOS-N software based on the NMR chemical shifts. Bar height corresponds to the confidence value and bar color indicates the predicted type of spatial structure.

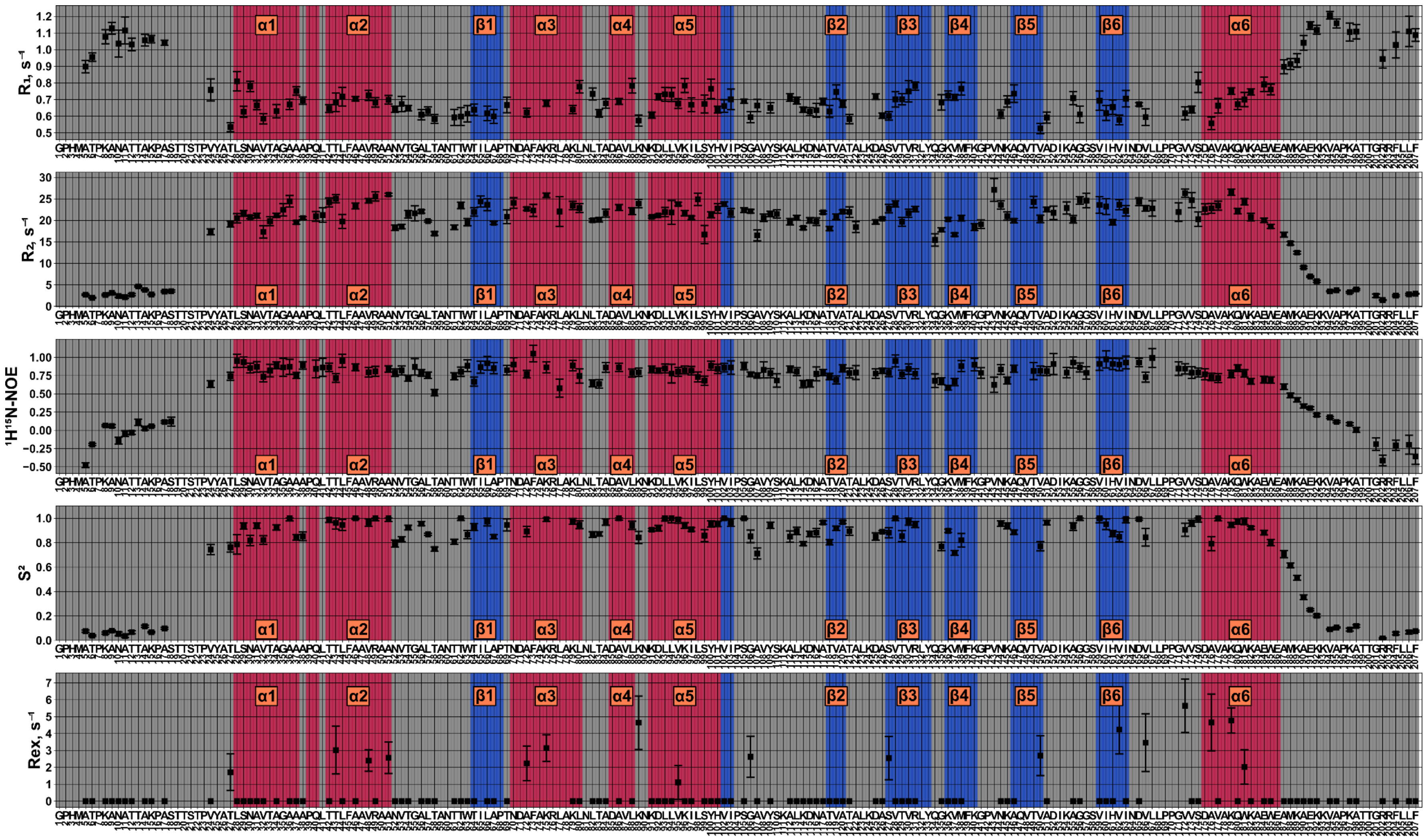

Supplementary Figure 7. Per-residue dynamics of the AstaPo1/AXT complex. NMR relaxation parameters of ^{15}N nuclei (rates of longitudinal (R_1) and transverse (R_2) relaxation, heteronuclear equilibrium NOE (^{1}H,^{15}N NOE)) and internal mobility parameters - generalized order parameter S^2 and exchange contribution to the transverse relaxation R_{ex} measured for AstaPo1 in complex with AXT. The elements of the secondary structure are indicated by red (helices) and blue (strands) bars.

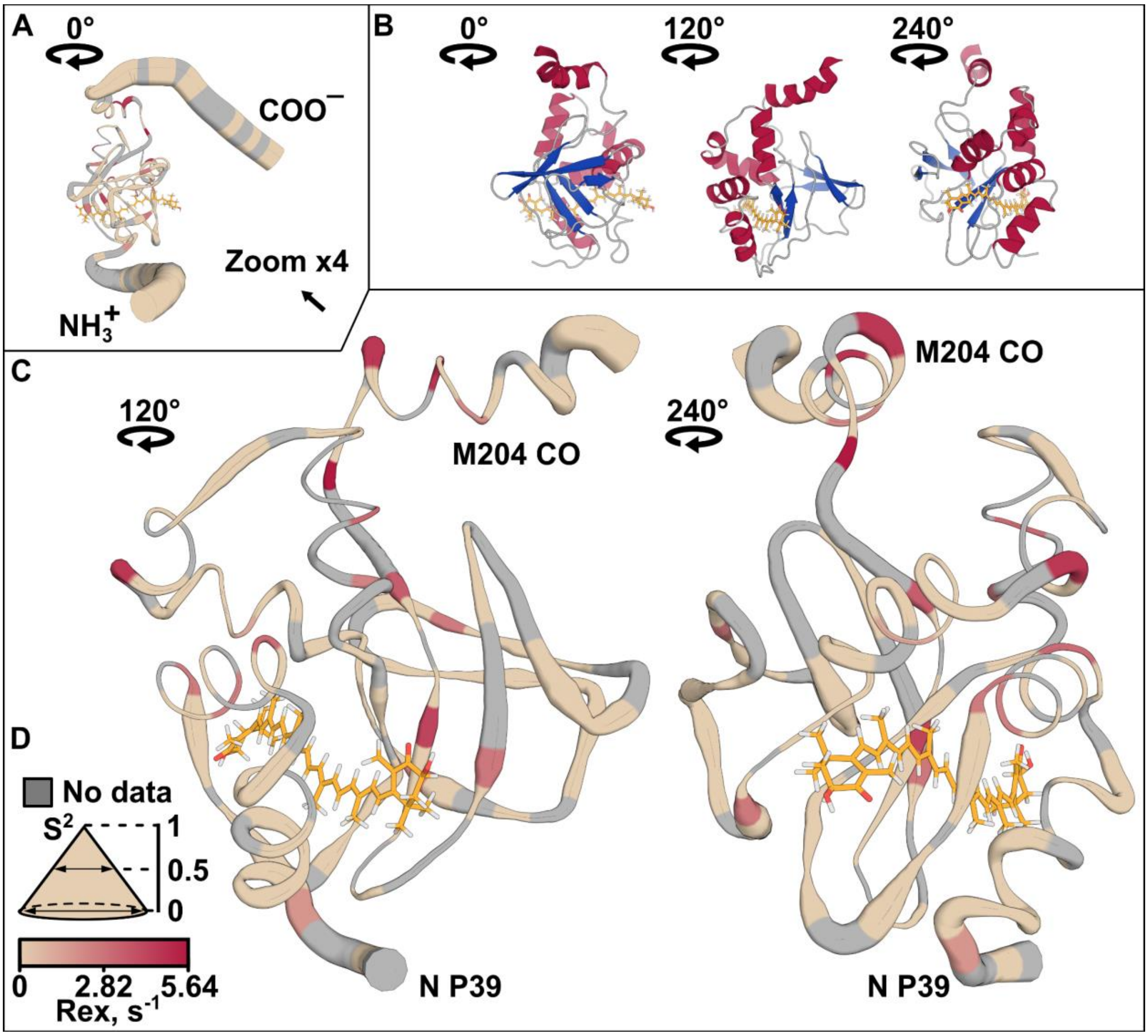

Supplementary Figure 8. Dynamics of the AstaPo1(AXT) structure. **A,C** - "sausage plot", highlighting the dynamics of the AstaPo1/AXT backbone. The thickness of the ribbons is inversely proportional to the generalized order parameters (S^2) of HN bonds. Residues with significant contributions of slow motions to the transverse relaxation (Rex) are colored red, according to the displayed scale. Grey regions correspond to the residues with no data available (prolines, overlapped, and too broad signals). Mobile terminal regions are removed on panel **C** for clarity. Panel **B** displays the cartoon of the views shown in **A** and **C**, denoted as 0, 120 and 240°. **D** - indication of ribbon color and thickness dependence in panel **C** on the intramolecular dynamic parameters of AstaPo1 backbone (S2 and Rex).

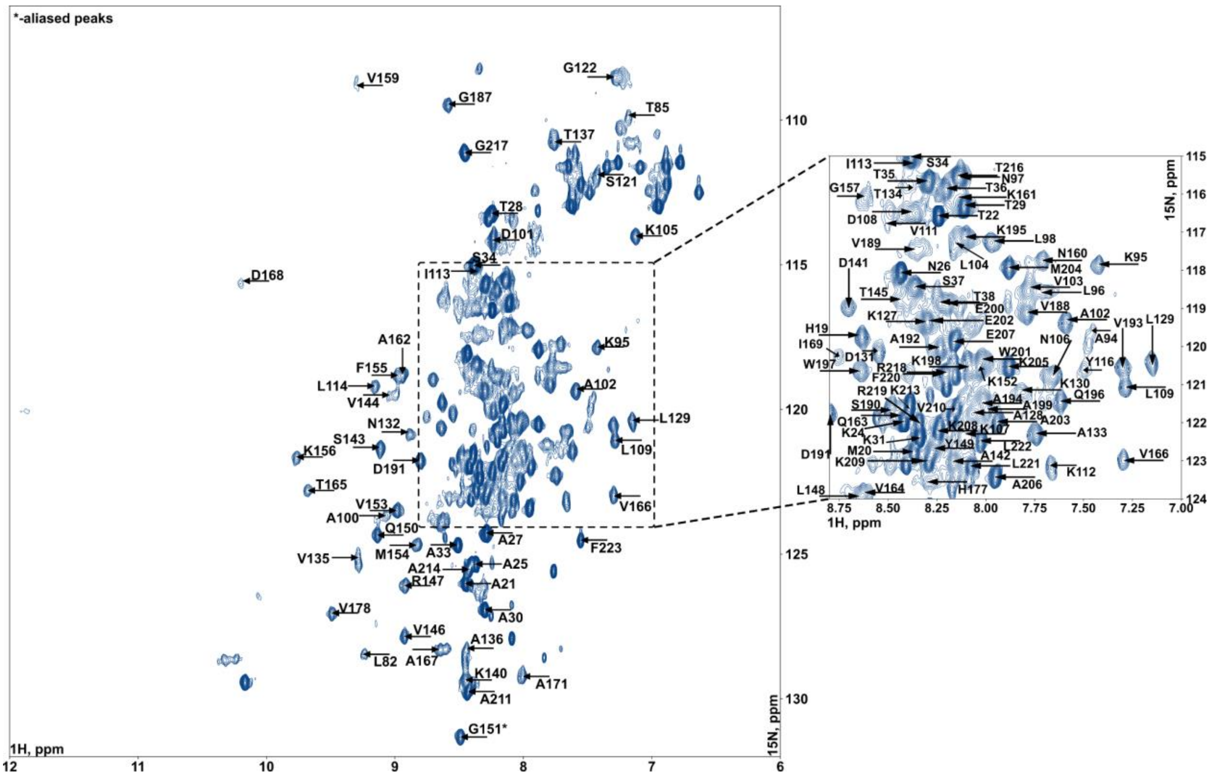

Supplementary Figure 9. $^{1}H^{15}N$-HSQC spectrum of AstaPo1(apo). T=298K, pH=6.0. Positions of aliased peaks are highlighted with asterisks.

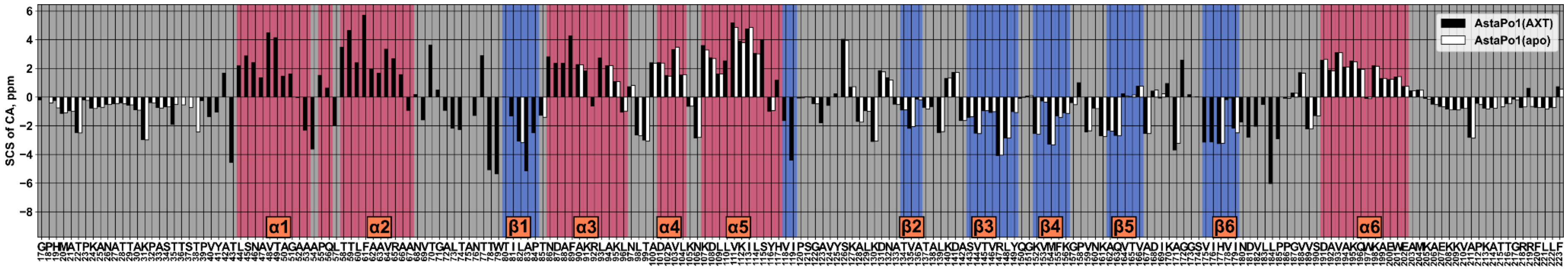

Supplementary Figure 10. Comparison of [13]Cα secondary chemical shifts (SCS) between AstaPo1(AXT) (black color) and AstaPo1(apo) (white color).

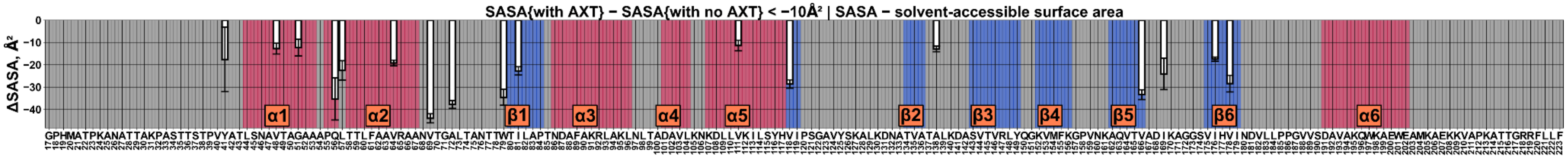

Supplementary Figure 11. Solvent-accessible surface area (SASA) changes with and without carotenoid using 10 A^2 as cutoff.

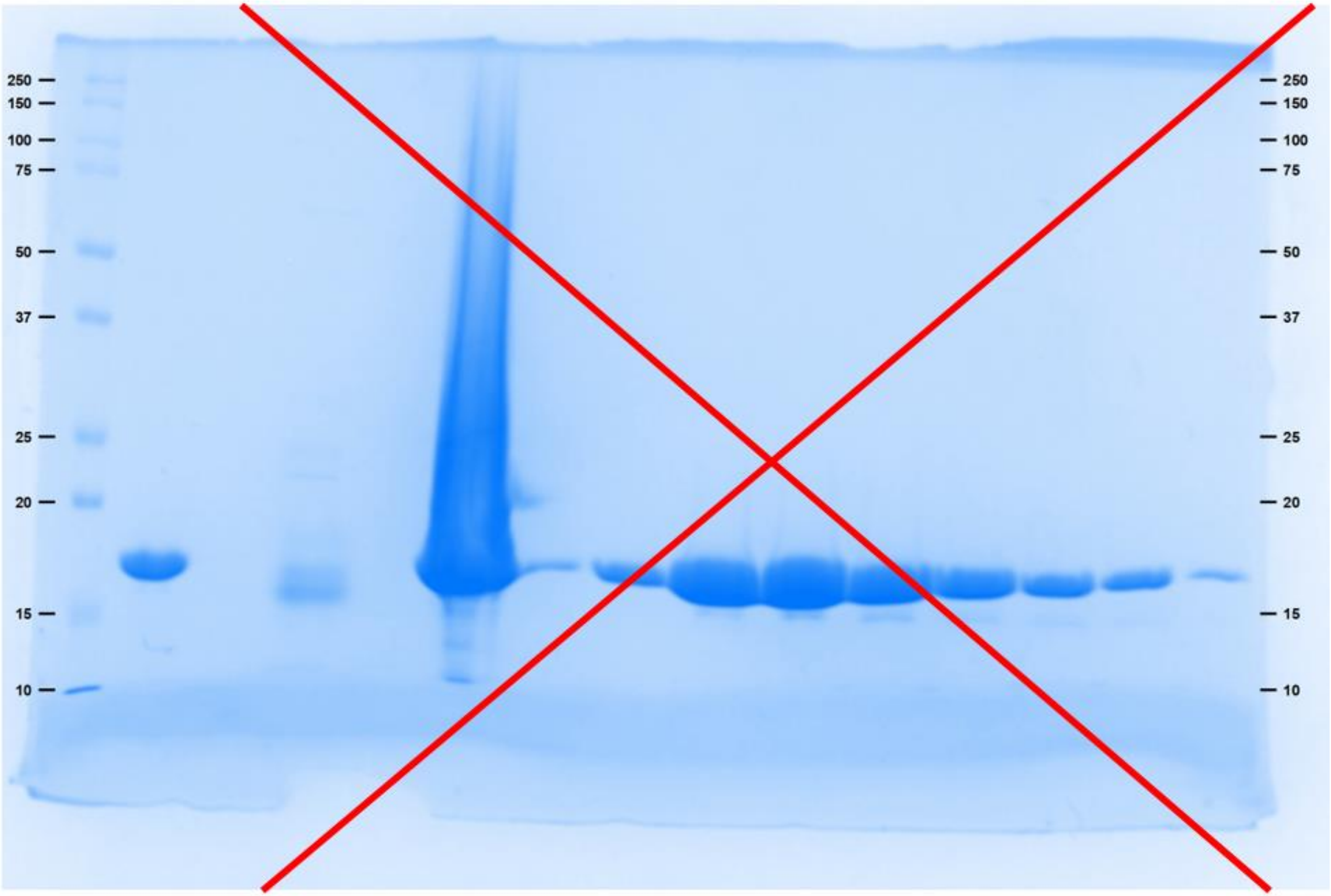

Supplementary Figure 12. Uncropped gel presented in Fig. 5e.
